# A collaborative opioid prescribing (CoOP) model linking opioid treatment programs with office-based buprenorphine providers

**DOI:** 10.1186/1940-0640-10-S1-A63

**Published:** 2015-02-20

**Authors:** Kenneth B Stoller

**Affiliations:** 1 Department of Psychiatry and Behavioral Sciences, Johns Hopkins University School of Medicine, Baltimore, MD, 21205, USA

## Background

Despite abundant research demonstrating good efficacy, pharmacotherapies for substance use disorders are underutilized. Buprenorphine holds promise to significantly increase the availability of medication-assisted therapy for opioid use disorder; yet despite dramatic increases in overdose deaths driven largely by prescription opioid use, only about half of DATA-waivered physicians prescribe buprenorphine.

## Methods

This presentation describes a collaborative care model in an addiction treatment program with an opioid treatment program component partnered with community medical practices in order to: 1) increase the willingness of primary care physicians to deliver office-based opioid treatment (OBOT) with buprenorphine; and 2) enhance the quality of care for patients receiving OBOT services. A collaborative opioid prescribing (CoOP) model was devised, linking primary care sites with an addiction treatment program (Johns Hopkins Broadway Center; JHBC), such that JHBC provided the initial assessment, medication induction/stabilization, and ongoing counseling, while the primary care sites provided OBOT. An adaptive stepped-care model adjusts counseling attendance and medication prescribing/dispensing based on ongoing indicators of treatment response. When necessary, medication dispensing is shifted between the OBOT and JHBC dispensary sites, and care is coordinated during the entire treatment episode. Early evaluation of this model has been very promising.

## Results

Since 2009, 81 JHBC patients received OBOT from a total of 22 providers. Twenty percent of these patients were admitted to the JHBC with existing OBOT providers, while the remainder were referred to a provider by the program. Sixty-two percent of these patients were male, 62 percent were Caucasian, and 38 percent were African American. Four percent were ages 18–24, 47 percent were 25–44, and 49 percent were 45–64. CoOP model appears to be a feasible approach to increase the availability and efficacy of office-based buprenorphine maintenance through collaboration between opioid treatment programs and office-based providers. Provision of concurrent psychosocial treatment, collaborative stepped care, and expert consultation by the treatment program can enhance OBOT services.

## Conclusions

Facilitation of collaborative systems such as the CoOP model may be a powerful strategy to increase availability and efficacy of mediation-assisted therapy for opioid use disorder.

